# Surgical outcomes of three different weakening procedures of inferior oblique muscle in the treatment of unilateral superior oblique palsy

**DOI:** 10.1186/s12886-020-01568-w

**Published:** 2020-07-20

**Authors:** Mohamed F. Farid, Mohamed Anany, Marwa Abdelshafy

**Affiliations:** Department of ophthalmology Benha University, 1 El Amira Fawzya st., El Vilal, Benha, Egypt

**Keywords:** Superior oblique palsy, Inferior oblique myectomy, Inferior oblique anterior transposition, Inferior oblique combined resection-anterior transposition

## Abstract

**Background:**

To compare surgical outcomes and complications of three inferior oblique weakening procedures; Inferior Oblique Myectomy (IOM), Inferior Oblique combined Resection-Anterior Transposition (IORAT) and Inferior Oblique Anterior Transposition (IOAT) in the management of unilateral Superior Oblique (SO) palsy.

**Methods:**

Retrospective review of medical records of all patients with unilateral SO palsy who underwent one of the aforementioned IO weakening procedures at Benha University hospital was performed. Patients were excluded if surgery was bilateral or combined with other vertical muscle surgery. Primary outcome parameters were improvement of Hypertropia (HT) in primary gaze, side gazes, on alternate head turn, Inferior Oblique Overaction (IOOA), Superior Oblique Underaction (SOUA), correction of head tilt and postoperative complications.

**Results:**

The review reveals a total of 65 patients with unilateral SO palsy; 54 congenital and 11 acquired, who met the study criteria and were classified into 3 groups; IOM group (24cases), IORAT group (19cases) and IOAT group (22cases). Compared with IOM, both IORAT and IOAT induced significant correction of HT in primary position, ipsilateral gaze, contralateral head tilt and IOOA. IORAT was significantly more effective than IOAT in correction of HT in ipsilateral gaze and contralateral head tilt while there was no statistical difference between the three groups in correction of HT in ipsilateral gaze, contralateral head tilt and SOUA. Postoperative Anti-elevation was significantly recorded following IORAT (6 cases, 31%) than IOAT (3 cases, 13%) and IOM (one cases, 4%).

**Conclusions:**

The IORAT and IOAT were more superior to IOM in correction of IOOA and HT in the primary position and some other gaze positions. However, superiority of IORAT over the other two procedures should be weighed against its significant association with postoperative underaction of IO muscle and anti-elevation syndrome.

## Background

Superior oblique (SO) palsy, either congenital or acquired is the most common form of isolated cranial nerve palsy, and its unilateral involvement is more common than the bilateral one [1]. Diagnosis is often made by Parks-Bielchowksy three-step test, in which hypertropia (HT) increases on adduction of the involved eye and on ipsilateral head tilt. Other presenting features include diplopia, anomalous head tilt and asthenopia [2, 3]. Wide varieties of surgical strategies have been proposed to treat unilateral SO palsy and they include strengthening of ipsilateral weak SO muscle (by tucking or advancement), weakening of ipsilateral overacting inferior oblique (IO) muscle, recession of the contralateral inferior rectus muscle or ipsilateral superior rectus muscle [4]. However, weakening of the ipsilateral overacting IO muscle is currently the most common surgical procedure performed. Weakening procedures of IO muscle have been evolved over years and they include disinsertion, denervation-extirpation, myectomy and recession [5]. Elliott and Nankin in 1982 [6] suggested that anterior transposition of IO muscle would increase its depressor effect, though this could be penalized by development of defective ocular elevation (anti elevation syndrome).

Recently, augmentation of IO anterior transposition procedure by resection of a segment of IO muscle was postulated to enhance the depressor effect of IO anterior transposition procedure [7]. In the current study, we compared the effect of three IO weakening procedures, myectomy,anterior transposition and combined resection-anterior transposition, in correction of HT in different positions of gaze as well as on degree of oblique muscle dysfunction in unilateral SO palsy.

## Methods

This study was conducted according to the principles of the Declaration of Helsinki and was approved by the Ethics Committee of Benha university hospital affiliated to Benha University. The medical records of consecutive patients with unilateral SO muscle palsy who were operated by single surgeon (MFF) at Benha University Hospital were retrospectively reviewed. Patients who were operated by weakening of IO muscle using either myectomy, anterior transposition or combined resection-anterior transposition of inferior oblique muscle were included. Patients were excluded if surgery was bilateral, combined with other vertical muscle surgery or if they had a history of previous strabismus surgery. Follow-up of at least 6 months after surgery was required. Patients with incomplete data or missed follow up were also excluded. Weakening of IO muscle by any of the aforementioned procedures was performed if HT was maximum in contralateral upgaze which denotes a major role of IO overaction (IOOA) as a contributing factor to the HT. Cases in which HT was maximum in contralateral downgaze were treated by tucking the SO muscle and were not included in this study.

Diagnosis of unilateral SO palsy was made by the presence of primary position HT which increased on adduction of the involved eye and on tilting the head to the same side of involvement (positive Parks-Bielchowsky Three-Step Test) [2, 3].Congenital cases were identified by the long duration of head tilt, presence of facial asymmetry and photographic documentation of head tilt in childhood photographs. Acquired cases were diagnosed by the lack of the aforementioned signs and by the presence of cyclovertical diplopia with an antecedent event related to its onset such as head trauma or CNS disease [8, 9]. The amount of HT in cardinal positions and in head tilts to the right and left was measured by the prism and alternate cover test (PACT) preoperatively and at each postoperative visit. Ocular motility was assessed using a − 4 to + 4 scale and overaction of the ipsilateral IO muscle (IOOA) and underaction of ipsilateral SO muscle (SOUA) was documented before operation and at each postsurgical visit. The severity of IOOA was graded in adduction by comparing the height difference between both eyes at the 6 o’clock limbus and graded according to the amount of overelevation of the affected eye relative to the normal one; (+ 1): 1 mm difference, (+ 2): 2 mm, (+ 3): 3 mm and (+ 4): 4 mm difference between both eyes. Using the same principle, SOUA was assessed in down and in position and was graded by comparing the height difference at 12 o’clock limbus according to the amount of under depression of the affected eye (relative to the normal eye) as follows: (− 1): 1 mm difference, (− 2): 2 mm, (− 3): 3 mm and (− 4): 4 mm difference between depression of both eyes. The presence of head tilt was documented pre and postoperatively and any postoperative change in the head tilt was documented as; a, complete resolution; b, reduced but not completely resolved; c, not changed. Improvement of the head tilt after operation was confirmed by comparing preoperative and postoperative photographs.

Informed written consent for the surgical procedures was obtained from all patients or parents/guardians of the children before surgery and all procedures were performed by the senior author (MFF)under general anesthesia. In the three groups, IO muscle was reached through inferior temporal conjunctival incision. After hooking of the muscle and lyses of its facial attachments, the IO muscle was clamped at its insertion by artery forceps and then severed from the sclera. In the myectomy group (IOM), another forceps was applied 6-8 mm from the first one and the segment in-between was removed after cauterizing the muscle ends. In the combined resection-anteriorization group (IORAT), a segment of 4 mm was measured from the first hemostat and a double armed 6/0 Vicryl suture was applied to secure the muscle at that distance with locking bites at each edge of the muscle. After the 4 mm segment was resected, the muscle was anteriorly transposed and fixed to the sclera immediately adjacent to the lateral border and exactly at the level of the inferior rectus insertion with temporal spreading of IO posterior fibers was limited to 2-3 mm [7]. In the anteriorization group (IOAT), the Vicryl suture was applied to the severed tendon without resection and the muscle was anteriorly transposed and sutured to the sclera immediately adjacent to the lateral border of the inferior rectus insertion with limited temporal spreading of posterior fibers.

Outcome measures include effect of each procedure on HT in different positions of gaze as well as on oblique muscles’ dysfunction. Findings at the last postoperative visit were used in the analysis of the surgical results. The collected data were tabulated and analyzed using SPSS version 16 software (SPSS Inc., Chicago, ILL Company). Categorical data were presented as numbers and percentages and were analysed by Chi square (χ^2^) and Fisher’s exact tests. Quantitative data were tested for normality using Shapiro-Wilks test assuming normality at *P* > 0.05. Normally distributed variables were expressed as mean ± standard deviation and analyzed by one-way analysis of variance (ANOVA), while non parametric data were presented as median and inter-quartile range (IQR), and were analyzed by Kruskal Wallis (KW) test. Significant ANOVA or KW tests were followed by post hoc multiple comparisons using Bonferroni adjusted tests to detect significant pairs. A *p* value less than or equal to 0.05 was considered significant.

## Results

The data of 65 patients with unilateral SO palsy met the inclusion criteria and were included in the study. The first group comprises 24 patients (36.9%) and they underwent unilateral IO myectomy (IOM group), the second group (IORAT) includes 19 patients (29.2%) who underwent IO combined resection and anterior-transposition while the third group comprises 22 patients (33.8%) who underwent IO anterior transposition (IOAT group). Average (median, interquartile range) follow up for all patients was 8(7–13) months (Table 1).

**Table 1:** Patient Characteristics and surgical complications

Parameter	IOM (no.24)	IORAT (no.19)	IOAT (no.22)	***P***value
**Age at time of surgery (years) Median (IQR)**	11.0 (7.3–19.5)	12.0 (8.0–15.0)	10.0 (6.8–11.5)	0.36†
**Length of follow up (months) Median (IQR)**	7.0 (6.0–12.75)	9.0 (8.0–13.0)	8.5 (7.0–13.0)	0.34 †
**Right /left eye**	14/10	13/6	17/5	0.39 *
**Male/female gender**	13/11	11/8	6/16	0.089*
**Congenital/acquired**	19/5	16/3	19/3	0.91 ^**F**^
**Pre-op head tilt (no., %)**	21 (87.5)	16 (84.2)	19 (86.4)	1.0 ^**F**^
**Residual post-op head tilt (no., %)**	13 (61.9)	9 (56.2)	12 (63.1)	0..907*
**Anti-elevation syndrome (no., %)**	1 (4%)	6(31%)	3 (13%)	< 0.045* §

*IOM* inferior oblique myectomy group, *IORAT* inferior oblique combined resection-anterior transposition group, *IOAT* inferior oblique anterior transposition, *IQR* inter quartile range, *no.* number of cases

† *P* value by Kruskal Wallis test, * *P* value by Pearson Chi-Square, **F;***p* value of Fisher’s exact test,§; significant *p* value

Table 2 shows comparison between different study parameters in the three groups. Although preoperative HT in the primary position and contralateral gaze was considerably larger in IORAT and IOAT groups relative to IOM group, however, there was no statistically significant difference between different study parameters before surgery in all groups. Regarding correction of HT in different gaze positions after surgery, IORAT and IOAT procedures attained significantly more improvement of primary position HT (*p* < 0.001) and IOOA (*p* = 0.001) relative to IOM, and further analysis by Bonferroni post hoc test showed no statistically significant difference between IOAT and IORAT groups. Similarly, IORAT and IOAT procedures attained a significant improvement of HT in contralateral gaze (*p* < 0.001) and ipsilateral head tilt (*p* < 0.001), but further analysis by Bonferroni post hoc test revealed more significant correction in favor of IORAT group. However, there was no statistically significant difference between the three procedure in correction of HT in ipsilateral gaze (*p* = 0.23), contralateral head tilt (*p* = 0.103) and SOUA (*p* = 0.96) (Table 2).

**Table 2:** Comparison of different study’s parameters between the three groups both pre and postoperatively

Item [Median (IQR)]	Preoperative				Postoperative				Improvement			
	IOM (*N* = 24)	IORAT (*N* = 19)	IOAT (*N* = 22)	***P***-value	IOM (*N* = 24)	IORAT (*N* = 19)	IOAT (*N* = 22)	***P***-value†	IOM (***N*** = 24)	IORAT (***N*** = 19)	IOAT (***N*** = 22)	***P***-value
**HT PP**	21.1 ± 6.2◊	25.1 ± 8.5	26.2 ± 7.8	0.063^≠^	7.0 (5–11)	2.0^a^ (0.0–8.0)	6.0 (4–10)	0.021 §	12.9 ± 3.6	20.3 ± 4.2^**a**^	19.5 ± 5.7^**a**^	< 0.001^**≠**^**§§**
**HT contra gaze**	33.7 ± 7.2◊	39.7 ± 10.3	38.5 ± 9.99	0.075^≠^	12.0 (8–15)	8.0 ^b^ (4–14)	12.0 (7.5–18.5)	0.058	22.0 (18–25)	31.0 ^**ab**^ (28–32)	25.0^a^ (22.0–27.3)	< 0.001**†§§**
**HT Ipsi gaze**	10.0 (8–10)	6.0 (4–14)	11.0 (6–15)	0.081**†**	3.0 (2–4)	0.0 (−2.0–4)	4.0 (0.0–8)	0.063	6.0 (5–8)	6.0 (6–9)	7.0 (6–8)	0.23**†**
**HT Ipsi tilt**	39.5 ± 7.8◊	43.9 ± 14.2	41.5 ± 10.7	0.42^≠^	18.0 (12–25)	7.0 ^ab^ (0.0–15)	12.0 (7.5–18)	< 0.001§§	21.5 ± 3.8	36.9 ± 8.0^**ab**^	27.8 ± 2.9^**a**^	< 0.001^**≠**^**§§**
**HT contra tilt**	9.0 (5.3–15)	8.0 (5–10)	10.0 (7.5–12)	0.35**†**	1.5 (0.0–6.0)	0.0 ^a^ (−2.0–2.0)	2.0 (−2.0–4.0)	0.018§	8.0 (5.3–9.0)	8.0 ^**a**^ (8–10)	8.0^**a**^ (7–10)	0.103 **†**
**IOOA**	2.0 (2–3)	2.0 (2–3)	3.0 (2–3)	0.14**†**	0.0 (0–0)	−1.0 ^a^ (−1.0–0.0)	0.0 (0–0)	0.004 §	2.0 (1–3)	3.0 ^**a**^ (3–3)	3.0^**a**^ (2–3)	0.001**†§§**
**SOUA**	− 2.0 [−3.0- (−1.0)]	−2.0 [−3.0-(− 1.0)]	−2.0 [− 2.0- (− 1.0)]	0.69**†**	0.0 (0–0)	0.0 (− 1.0–0)	0.0 (0–0)	0.077	2.0 (1–2)	1.0 (1–2)	2.0 (1–2)	0.96**†**

*HT PP* Hypertropia in primary position measured in prism diopter, *IOOA* inferior oblique muscle overaction, *SOUA* superior oblique muscle underaction, *IQR* inter quartile range, **◊**, data was presented as mean ± Sd, **≠**; *p* value by ANOVA, **†**; *p* values by Kruskal Wallis (KW) test, §; Significant *p* value, §§: highly significant *p* value

Significant KW test was followed by post hoc multiple comparisons using Bonferroni adjusted tests to detect the significant pairs: ^a^; significance in comparison with IOM group, ^b^ significance in comparison with IOAT group

In all groups, all patients with preoperative head tilt responded positively to either procedure as there was no recorded cases of non-improvement of head tilt in each of the study groups. In IOM group, there were 21 patients with preoperative head tilt, which was completely resolved by the surgical procedure in 8 patients (38%) and was improved but not eliminated in 13 patients (61.9%). In IORAT group and out of the 16 patients who had head tilt before surgery, the procedure induced complete resolution of the head tilt in 7 cases (43.7%) and significant improvement in the remaining 9 cases (56.2%). The IOAT caused disappearance of the head tilt in 7 out of 19 cases with preoperative head tilt (36.8%) with improvement in the remaining 12 cases (63.1%). (Table 1) When the effect of either procedure on preoperative head tilt was analyzed, the difference was found statistically insignificant (*P* = 0.907).

At the last postoperative visit, persistent defective elevation, mainly in abduction (anti-elevation syndrome) [8, 9] was recorded in 1, 6 and 3 cases in IOM, IORAT and IOAT groups respectively (*P* = 0.045). In all those cases, there was no hypotropia in the primary position but only in upgaze (mean 8.3 ± 3.5 PD). All patients who showed significant persistent anti-elevation syndrome were offered corrective surgery but only three cases accepted (two cases from IORAT and one case from IOAT groups). These cases were treated by recessing the previously anteriorized IO muscle to a point 4 mm posterior to inferior rectus muscle insertion as suggested by Kushner [10] which resulted in significant improvement in the limitation of elevation in all those patients without any recurrence of either IOOA or head tilt. No other intra or postoperative complication was recorded in any of the study groups.

## Discussion

In this retrospective study, three different IO weakening procedures were compared in the treatment of unilateral SO palsy in terms of effect of each procedure on HT in different positions of gaze, anomalous head tilt, oblique muscles’ dysfunction and postoperative complications. In general, and relative to IOM, we have found that both IORAT and IOAT procedures attained significant improvement of HT in primary position, contralateral gaze, ipsilateral head tilt and IOOA with more significance of IORAT in correction of HT in contralateral gaze and ipsilateral head tilt. In addition, we have also found that all procedures were equally effective in improving SO underaction and HT in both ipsilateral gaze and contralateral head tilt. However, significant higher rates of postoperative anti-elevation secondary to IORAT could complicate its use in the management of unilateral superior oblique palsy.

Weakening of overacting IO muscle has been widely considered as the first surgical option in treatment of symptomatic SO palsy, and this is because its effectiveness in alleviation of ocular deviation and abnormal head positions while avoiding adverse side effects associated with other forms of strabismus surgeries such as iatrogenic Brown syndrome and overcorrections [1, 11].In previous reports concerning IOM, the average correction of HT in primary position ranged from 12.5 to 14 PD and unit correction of IOOA ranged from 2 to 2.4 units with favorable outcomes regarding correction of AHP and symptomatic diplopia [12–14]. These results closely match ours in the IOM group where there is an average correction of HT in the primary position and IOOA by 13 PD and 2.0 units, respectively.

IOAT has been advocated as an effective weakening procedure for controlling IOOA associated with SO palsy [15, 16]. In previous reports, the range of average correction of HT was 9.5 to 19.2 PD in the primary position and 17 to 27.7 PD in contralateral gaze, while average correction of IOOA ranged between 2.5 to 4 units [15–19]. In the current study, IOAT achieved an average 20.7 PD correction of HT in the primary position and 30 PD correction in contralateral gaze, while IOOA was improved by an average of 3 units. The incidence of postoperative anti-elevation syndrome following IOAT was reported to range between 0 and 27% [6, 15–19] while in the current trial, the incidence of anti-elevation syndrome following IOAT was 13%.

Farvardin and colleagues have reported satisfactory results of combined resection and anterior transposition of IO muscle in cases of dissociated vertical deviation associated with IOOA. They postulated that segment resection of the anteriorly transposed IO muscle augments its anti-elevating effect [7]. Later, they studied the effect of the combined procedure on 27 unilateral SO palsy patients with large (20-25 PD) HT in the primary position, and have found that the procedure improved HT in the primary position and in contralateral gaze by an average of 21.2 PD and 27.8 PD respectively with development of mild defective elevation in only one case [20] These results are comparable to ours whereas the IORAT procedure improved HT in the primary position and in contralateral gaze by an average of 23.7 PD and 36 PD respectively. However, the IORAT procedure in our study induced a significant number of postoperative anti-elevation syndrome (6 out of 19 cases, 31%).

We believe that the present study is the first to specifically compare three different IO weakening procedures in the treatment of SO palsy. Of note, there were some previous reports which compared three different IO weakening procedures, but those studies were mainly concerning IOOA secondary to horizontal strabismus [21, 22]. In contrast to the current study which evaluates the effect of each procedure on HT in all gaze positions as well as oblique muscle dysfunction, outcome measures of most of the previous reports were mainly concerning procedure effect on IOOA and HT in the primary position. Few studies concerned HT in contralateral gaze [14, 20] and SOUA [14], and none up to our knowledge, have evaluated the result of a procedure on HT in all relevant gaze positions, as well as IOOA and SOUA.

## Conclusions

Limitations of this study include its retrospective nature, small sample size, limited follow up period (average; 8 months) and non-assessment of vertical fusional amplitude. However, this study provides valuable insights about characteristics of three different weakening procedures in treatment of SO palsy. IORAT and IOAT were significantly superior to IOM in correction of HT in primary position and IOOA. In contralateral gaze and ipsilateral head tilt, both procedures were also significant relative to IOM but with more significance in favor IORAT. There was no significant difference between the three procedures in correction of HT in ipsilateral gaze, contralateral head tilt and SOUA. However, and compared with IOM and IOAT, superiority of IORAT should be weighed against its significant association with persistent IO underaction and defective ocular elevation, which could hinder its use in management of unilateral superior oblique palsy.

## Data Availability

The datasets used and/or analysed during the current study are available from the corresponding author on reasonable request.

## Abbreviations

IOM: Inferior Oblique Myectomy
IORAT: Inferior Oblique Combined Resection-Anterior Transposition
IOAT: Inferior Oblique Anterior Transposition
SO: Superior Oblique
IO: Inferior Oblique
HT: Hypertropia
IOOA: Inferior Oblique Overaction
SOUA: Superior Oblique Underaction
